# Acute and post-acute sequelae of SARS-CoV-2 infection: a review of risk factors and social determinants

**DOI:** 10.1186/s12985-023-02061-8

**Published:** 2023-06-16

**Authors:** Chumeng Wang, Akshara Ramasamy, Monica Verduzco-Gutierrez, W. Michael Brode, Esther Melamed

**Affiliations:** 1 Department of Neurology, Dell Medical School, University of Texas at Austin, Austin, TX USA; 2 Department of Physical Medicine and Rehabilitation, University of Texas at San Antonio, San Antonio, TX USA; 3 Department of Internal Medicine, Dell Medical School, University of Texas at Austin, Austin, TX USA

**Keywords:** COVID-19, PASC, Long-COVID, Risk factors, Social determinants

## Abstract

SARS-CoV-2 infection leading to Coronavirus Disease 2019 (COVID-19) has caused more than 762 million infections worldwide, with 10–30% of patients suffering from post-acute sequelae of SARS-CoV-2 infections (PASC). Initially thought to primarily affect the respiratory system, it is now known that SARS-CoV-2 infection and PASC can cause dysfunction in multiple organs, both during the acute and chronic stages of infection. There are also multiple risk factors that may predispose patients to worse outcomes from acute SARS-CoV-2 infection and contribute to PASC, including genetics, sex differences, age, reactivation of chronic viruses such as Epstein Barr Virus (EBV), gut microbiome dysbiosis, and behavioral and lifestyle factors, including patients’ diet, alcohol use, smoking, exercise, and sleep patterns. In addition, there are important social determinants of health, such as race and ethnicity, barriers to health equity, differential cultural perspectives and biases that influence patients’ access to health services and disease outcomes from acute COVID-19 and PASC. Here, we review risk factors in acute SARS-CoV-2 infection and PASC and highlight social determinants of health and their impact on patients affected with acute and chronic sequelae of COVID-19.

## Background

The COVID-19 pandemic has led to over 762 million infections and 6.8 million deaths [1]. Individuals may experience asymptomatic, mild, severe or fatal illness with symptoms ranging from fever, runny nose, cough, dyspnea, fatigue, diarrhea, headache or multi-organ failure, and lasting on average for one to four weeks [2].

A significant portion of COVID-19 patients, estimated at 10–30% (over 10–30 million people in the US and over 60–180 million people worldwide), may experience long-term symptoms, known as post-acute sequelae of SARS-CoV-2 infection (PASC) or “Long-COVID.“ PASC symptoms can include fatigue, dyspnea, brain fog, chest and joint pain, and multi-organ dysfunction affecting the neurological, pulmonary, digestive, and cardiac systems [3, 4].

There is currently no widely accepted definition for PASC, with the World Health Organization defining PASC as “3 months from the onset of symptoms, lasting at least 2 months,” while the Centers for Disease Control and Prevention (CDC)’s guidance suggests “a wide range of new, returning, or ongoing health problems” experienced after infection with SARS-CoV-2 [3, 4].

Given the ongoing SARS-CoV-2 infections, there is a pressing need to not only understand and treat acute COVID-19 infection but to also better characterize PASC. Inclusivity and the diversity of affected patient views must be honored, and patient experiences should be translated into standardized studies investigating PASC to identify better therapies for the prevention and treatment of PASC.

The goal of our review is to compare the risk factors for acute SARS-CoV-2 infection and PASC as well as discuss how social determinants of health impact acute COVID-19 and PASC disease outcomes.

## Risk factors for acute COVID-19 and PASC

**Fig. 1:**
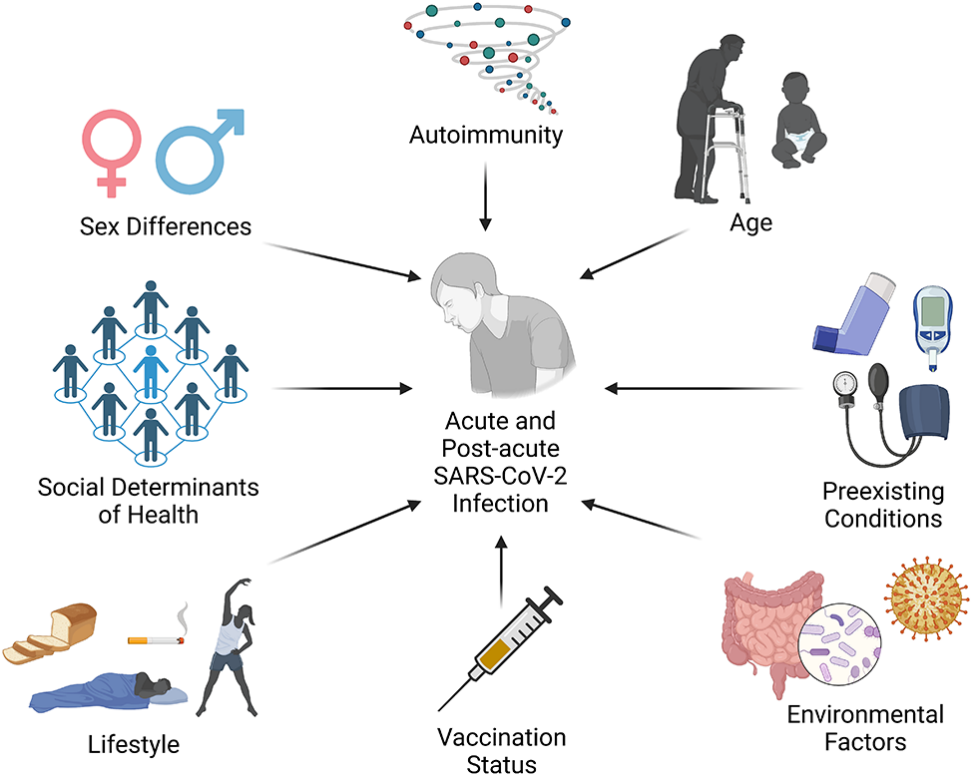
PASC Risk Factors. Multiple risk factors may contribute to an increased likelihood of developing PASC, including sex differences, autoimmunity, age, preexisting medical conditions, environmental factors such as gut microbiome dysregulation and EBV infection, vaccination status and lifestyle factors such as smoking, exercise, diet and alcohol, among other potential risk factors that continue to be evaluated. Additionally, social determinants of health serve as a modifying risk factor

The risk factors for acute COVID-19 and PASC continue to be elucidated. Here, we will review emerging data on risk factors for acute COVID-19 and PASC, including genetics, sex differences, age, co-morbid conditions, SARS-CoV-2 vaccines, as well as environmental, behavioral and lifestyle factors (Fig. 1).

### Genetic factors

#### HLA types

The Human Leukocyte Antigen (HLA) system facilitates immune regulation through the presentation of processed peptide antigens to T-cells [5]. Certain HLA haplotypes have been associated with a genetic predisposition to COVID-19 and may influence disease outcomes [5, 6]. For example, the HLA-B46:01114 haplotype has fewest binding sites for SARS-CoV-2, while the HLA-B15:03 haplotype has a greater ability to present conserved SARS-CoV-2 peptides. These findings suggest that individuals with the HLA-B*46:01114 haplotype may be at higher risk for more severe disease due to lower display of SARS-CoV-2 peptides to the immune system [6].

**Fig. 2:**
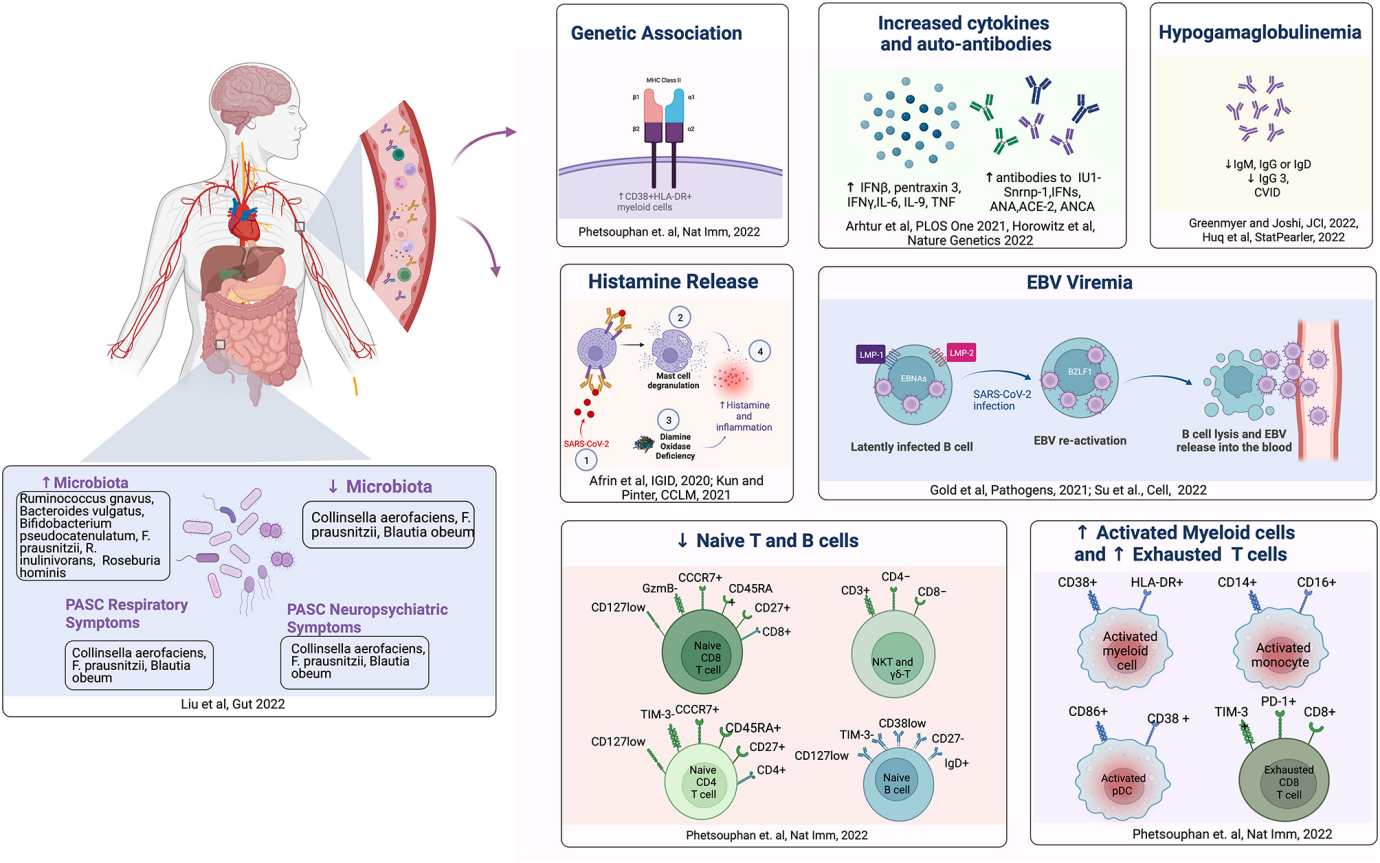
Genetic, Immune and Gut Microbiome Dysregulation in PASC. Patients with PASC display multifocal abnormalities in immune system activation and gut microbiome dysbiosis as well as specific genetic HLA associations. Within the innate and adaptive immune system, patients demonstrate decrease in naive T and B cells and immunoglobulins, and increase in activated myeloid cells, exhausted T-cells and autoantibodies. Patients may also have an increase in histamine release, which in combination with deaminase oxidase deficiency can contribute to augmentation of inflammation. SARS-CoV-2 may also potentially serve as a trigger in reactivating latent EBV, which could contribute to molecular mimicry-mediated ongoing PASC inflammation. Lastly, patients with PASC may have gut microbiome dysbiosis, that may correlate with neuropsychiatric and respiratory PASC symptoms

Type of HLA could also determine COVID-19 and PASC outcomes because of its role in triggering autoimmune reactions. For example, DRB1*15:01–DRB5*01:01–DQA1*01:02–DQB1*06:02 are overrepresented in patients with multiple sclerosis while HLA-DRB1*03, HLA-DRB1*15, and HLA-DRB1*04 are overrepresented in systemic lupus erythematosus (SLE) and dictate type of auto-antibodies and SLE symptoms [7, 8]. Interestingly, highly activated CD38^+^HLA-DR^+^ myeloid cells are elevated at 8 months in PASC patients compared to controls (Fig. 2) [9]. Subacute thyroiditis post-COVID has also been associated with presence of HLA-B*35 [10]. Additionally, different SARS-CoV-2 peptides may have varied immunogenicity at different HLAs, with HLA-B*40:01-presented ligands as most immunogenic [11]. Further research is necessary to determine which HLA haplotypes may better predict PASC in certain patients.

### Sex differences

Studies have found that biological sex is a significant risk factor in COVID-19. This is likely due to physiological differences between males and females that can affect the severity of infection and autoimmune responses [12–15].

#### Studies in males

Men have a higher risk of hospitalization and death from COVID-19, with one study showing an 18-fold higher in-hospital mortality risk [16]. In another study, over 89.8% of hospitalized males exhibited low testosterone levels [17]. However, evidence for testosterone in acute COVID-19 susceptibility has been controversial. For example, prostate cancer patients post androgen deprivation therapy (ADT) had a significantly reduced risk of COVID-19 than patients without ADT [18]. In contrast, elevated free testosterone was associated with higher COVID-19 severity in men [19].

In PASC, there has been a noted association with erectile dysfunction and decreased libido in males [20, 21]. Nevertheless, it remains unclear at this time whether male sex, hypogonadism (due to direct gonadal or hypothalamic SARS-CoV-2 damage leading to low testosterone) or other factors (e.g. depression, stress, immune dysregulation) may be responsible for the observed symptoms in men with PASC. Further research is needed to clarify male-specific risk factors such as hormones and genetic versus environmental risks for acute COVID-19 and PASC.

#### Studies in females

While males may be more vulnerable to acute COVID-19, females appear to be at a higher risk for PASC in a 4:1 female:male ratio [22, 23].

Interestingly, females may have different risk factors for acute COVID-19 and PASC depending on their hormonal status. For example, menopause has been associated with increased risk for severe acute COVID-19 illness, and estrogen augmentation was correlated with a lower risk of mortality from acute COVID-19 in postmenopausal women [24, 25]. Similarly, postmenopausal women have higher rates of acute COVID-19 infections compared to women pre- menopause, suggesting that estrogen may play a role in acute COVID-19 disease severity [26].

X chromosome genes have been implicated in sex differences in COVID-19 outcomes [27]. Notably, angiotensin converting enzyme 2 (ACE2), the principle receptor for SARS-CoV-2 entry into cells, is found on the X chromosome [27]. ACE2 expression is lower in the lungs of females compared to males, and estrogen downregulates ACE2 expression [28, 29]. Thus, lower ACE2 expression in females may potentially account for lower viral entry and lower severity of acute-COVID-19. Furthermore, Toll-like receptor 7 (TLR7), a regulator of interferon (IFN) production, is another X chromosome immune gene [30]. A higher dose of TLR7 expression has been suggested to lead to higher IFN signaling in acute COVID-19 and better viral clearance in females, though continued IFN signaling may lead to overactive immune activation and persistent inflammation, predisposing females to a higher autoimmunity risk and PASC [9, 31]. Further research is needed to fully understand the role of hormones and sex chromosome genes and how these factors contribute to male versus female specific risks in acute COVID-19 and PASC.

### Age

#### Age in acute COVID-19

Several studies have suggested that age is associated with acute COVID-19 severity, with older age emerging as an independent risk factor [32, 33].

In acute COVID-19, the risk of mortality increases with every five years of age [34]. Using an age-structured mathematical model, Davies et al. estimated clinical symptoms to appear in 21% of infections in 10- to 19-year-olds, increasing up to 69% of infections in adults over 70 [35].

#### Age in PASC

In PASC, the role of age has been controversial, with some studies finding age to be a significant predictor of PASC, with incidence rates from 9.9% in 18–49 year old patients to 21.9% in individuals over 70 [36]. Among women, age appears to be a female-specific risk factor with women aged 40–60 more susceptible to PASC [37, 38]. In contrast, other studies found that PASC risk decreases with age or has no association [39, 40]. Therefore, it is uncertain if age is an independent risk factor for PASC, and further research is needed to determine if certain age groups are more susceptible to PASC.

### Environmental, behavioral and lifestyle risk factors of acute SARS-CoV-2 infection and PASC

Environmental and behavioral risk factors are known to influence disease outcomes. In this section, we will focus on the reactivation of Epstein-Barr Virus (EBV), gut microbiome, and lifestyle factors in acute SARS-CoV-2 infection and PASC.

#### EBV reactivation

Over 90% of the global population harbors a latent EBV infection, which can stay dormant in B cells and reactivate under conditions of critical illness, stress, burns, immunocompromise, or other acute infections [41]. Primary EBV infection may be asymptomatic or associated with symptoms of mononucleosis, such as fatigue, fever, pharyngitis, cervical lymphadenopathy, and lymphocytosis [42]. EBV reactivation symptoms are typically experienced as a recrudescence of the primary infection symptoms, like fatigue, brain fog, sleep disturbances, arthralgias/myalgias, headaches, gastrointestinal complaints, and skin rashes [43].

#### EBV reactivation in acute COVID-19

Several studies have reported EBV reactivation in acute COVID-19. Chen et al. first reported that 55.2% of hospitalized COVID-19 patients tested positive for EBV IgM two weeks after disease onset [44]. Similarly, in a cohort of 104 COVID-19 Italian patients, Paolucci et al. found EBV reactivation in 95.2% of ICU patients [45]. In another study, 25% of COVID-19 patients had EBV reactivation and higher rates of mortality [46]. Since reactivation is common in critical illness (e.g. ICU patients with sepsis, burns, or pneumonia), further research on EBV reactivation in acute COVID-19 is necessary to determine whether treating EBV reactivation during acute COVID-19 illness may benefit patients long term in preventing SARS-CoV-2-related sequelae [47, 48].

#### EBV reactivation in PASC (Fig. 2)

In PASC, Gold et al. first reported that 66.7% of PASC patients and only 10% of the controls were positive for EBV reactivation, with a direct correlation between the number of PASC-related symptoms and presence of early antigen-diffuse immunoglobulin G antibody titers [49]. In analyzing the relationship of EBV viremia and SARS-CoV-2 RNAemia to PASC, Su et al. found that measurements of both EBV viremia and SARS-CoV-2 RNAemia at acute COVID-19 diagnosis were significantly correlated with later PASC-related memory problems [50]. Interestingly, PASC symptoms of fatigue and sputum production were exclusive to EBV viremia. Furthermore, Peluso et al. have also demonstrated that EBV reactivation may be a key factor in PASC and specifically relates to fatigue and neurologic symptoms [51]. These studies suggest that PASC symptoms may at least in part arise from EBV-induced damage and/or EBV-mediated immune dysregulation post SARS-CoV-2 infection.

Due to the noted EBV reactivation in acute SARS-CoV-2 infection and PASC, treatments used to alleviate symptoms in reactivated EBV in the absence of COVID-19 might also be useful to investigate as therapies to address EBV reactivation in acute COVID-19 and PASC. For example, reactivated EBV after hematopoietic cell transplantation has been treated with the anti-CD20 monoclonal antibody, rituximab [52]. Other antiviral therapies such as acyclovir, ganciclovir, and vidarabine inhibit viral DNA polymerase have been used to treat chronic active EBV, though have not been found to be effective in chronic non-active EBV [53]. Infusion of immunoglobulins (IVIG) is another promising therapy that has been successfully used in some critical patients without SARS-CoV-2 infection and may be beneficial in acute COVID-19 or PASC [54]. Further research is needed to determine whether the treatments used for non-SARS-CoV-2-related EBV reactivation may also be beneficial in the treatment of acute COVID-19 and PASC.

#### Gut microbiome

##### Gut microbiome studies in acute COVID-19

A growing body of research suggests that the gut microbiome composition is related to the severity of acute COVID-19 [55]. However, it is unknown whether any changes in the microbiome’s makeup occur after eradication of SARS-CoV-2.

##### Gut microbiome studies in PASC (Fig. 1)

A study of 106 patients found that PASC patients have significantly lower levels of *Collinsella aerofaciens*, *F. prausnitzii*, *Blautia obeum*, and a greater level of *Ruminococcus gnavus* and *Bacteroides vulgatus* than non-COVID-19 controls at six months [56]. Specific PASC symptoms may be associated with gut microbiome dysbiosis. For example, pathogens including *Streptococcus anginosus*, *Streptococcus vestibularis*, *Streptococcus gordonii* and *Clostridium disporicum* were correlated with persistent respiratory symptoms. Similarly, in patients with neuropsychiatric PASC, there was an association with the abundance of *Clostridium innocuum* and *Actinomyces naeslundii*. The relative abundance of *Bifidobacterium pseudocatenulatum, F. prausnitzii, R. inulinivorans, and Roseburia hominis*, known to benefit host immunity, exhibited the strongest inverse relationships with PASC.

#### Diet

##### Diet in acute COVID-19

Data on specific-diet outcomes in acute SARS-CoV-2 infection is currently limited. However, general guidelines for acute COVID-19 include a diet rich in vegetables, fruit, whole grains, healthy fats, low-fat dairy, and limiting red meat [57]. For example, a plant-based diet was linked to a decreased risk and severity of COVID-19 [58]. Similarly, participants following “plant-based” and “plant-based or pescatarian diets” had 73% and 59% lower odds of moderate-to-severe COVID-19 compared to individuals following “low carbohydrate, high protein diets,” who had 48% greater odds of moderate-to-severe COVID-19 [59].

##### Diet in PASC

In autoimmune diseases, balanced diets composed of whole grains, polyphenol-rich vegetables, and omega-3 fatty acid-rich foods may reduce inflammation and fatigue [60]. Whether an anti-inflammatory diet or supplements can be extended to PASC patients, thought to have immune dysregulation, is currently being studied, with over 20 trials listed on clinicaltrials.gov.

Nonetheless, one emerging diet gathering patient support is an anti-histamine diet, as overactivation of mast cells and histamine release may play a role in PASC (Fig. 1) [61, 62]. Histamine intolerance in PASC may be related to diamine oxidase decrease leading to mast cell activation syndrome [63]. Foods high in histamine include blue fish and fermented products such as cheeses, sausages, wine, beer, sauerkraut, and fermented soy derivatives [64]. The avoidance of such foods may constitute a low histamine diet, though current research is limited [65].

Increasing electrolyte, salt, and water intake may alleviate PASC-related-fatigue caused by autonomic dysfunction in PASC, notably Postural orthostatic tachycardia syndrome (POTS) [66]. Small, more frequent meals are recommended, and diets rich in fiber and probiotics may improve GI-related POTS symptoms.

Recently, a high-quality diet (upper 40% of Alternate Healthy Eating Index–2010 score) was found to be protective against PASC; however, considering the heterogeneity of PASC clinical presentations, future studies are needed to determine if specific dietary interventions can treat different PASC symptoms [67].

#### Alcohol

##### Alcohol in acute COVID-19

Alcohol abuse has been associated with an increase in acute lung injury and acute respiratory distress syndrome [68]. However, it is uncertain how alcohol consumption impacts COVID-19 risk, severity, and mortality.

Though Hamer et al. demonstrated no relationship between alcohol and acute COVID-19 hospitalization, Bailey et al. found that patients with alcohol use disorder had a greater risk of hospitalization and mortality [69, 70]. However, intake of spirits, beer and cider raised the risk of COVID-19 independent of consumed frequency or amount, while a low frequency of drinking wine and champaign (1–2 glasses/week) was protective against COVID-19, Although certain alcohols were related to decreased COVID-19, drinking cannot be deemed an effective mechanism for infection prevention.

##### Alcohol in PASC

High alcohol intake disrupts several pathophysiological pathways by increasing the levels of proinflammatory cytokines, disrupting alveolar macrophage activities in the lungs, and desensitizing respiratory ciliated cells [71–73]. As one of the PASC characteristics is prolonged inflammation, it is possible that chronic high-dose alcohol may further exacerbate inflammation by upregulating cytokines. However, it remains unclear how alcohol interacts with or contributes to PASC. Considering the stark increase in alcohol sales during the pandemic and dichotomy in current studies, further investigations are currently taking place, inclusive of the NIH RECOVER trial regarding alcohol and susceptibility to PASC [74].

#### Smoking

##### Smoking in acute COVID-19

There is inconclusive evidence surrounding the impact of smoking on the risk and severity of COVID-19. In one study, current smokers (71%) had 80% reduced probability of contracting COVID-19 than former smokers and nonsmokers [75]. Another study in 43,103 adults, found that patients reporting current smoking had a reduced incidence of hospitalization or death [76].

In contrast, current smokers showed greater risk of hospitalization and mortality when compared to never-smokers in a study by Clift et al. [77]. In a meta-analysis consisting of 46 peer-reviewed articles of 22,939 COVID-19 patients, of which 23.6% had disease progression, 12.7% had a history of smoking and 33.5% of prior-smokers reported illness progression, compared to 21.9% of nonsmokers [78]. Importantly, patients with a history of smoking or any tobacco use had an increased risk of COVID-19-related mortality [78].

##### Smoking in PASC

In comparison, emerging data suggest that smoking increases risk of PASC [79]. Specifically, smokers were more likely to experience tachycardia and/or high-blood-pressure. However, as with alcohol, the impact of smoking on PASC patients is still an area of active research and further studies are needed to confirm a causal relationship.

#### Exercise

##### Exercise in acute COVID-19

Following acute SARS-CoV-2 infection, people who exercise had improved clinical outcomes [80, 81]. With recovery from acute COVID-19, Udina et al. found small intervals of 30-min daily individualized therapeutic exercise intervention increased functional status post-ICU stay [82].

##### Exercise in PASC

The role of exercise in PASC management is controversial. Rebello et al. hypothesized that exercise mitigates the neuropsychiatric and endocrine consequences of PASC by stimulating the release of circulating factors that modulate the anti-inflammatory response, promote brain homeostasis, and enhance insulin sensitivity [83].

However, with symptoms such as fatigue and myalgias, PASC patients find it difficult to exercise. In a survey by Davis et al., 89.1% of PASC patients reported physical and/or mental post-exertional malaise [84]. In another study, PASC patients showed a significant decrease in peak exercise aerobic capacity, as well as an elevated hyperventilatory response during exercise [85]. Similarly, women with PASC exhibited an increased heart rate with exertion and heart rate recovery was delayed after a 6-minute walk test [86].

In a survey study, the majority of PASC patients (74.8%) claimed that physical activity worsened, 0.84% said their symptoms improved, some (20.9%) stated that it had a mixed impact, and 28.7% of participants said physical activity had no effect on their PASC symptoms [87], Similar to acute COVID-19 rehabilitation, current approaches to PASC recovery suggest a personalized rehabilitation approach by offering tailored exercises, starting with lower intensity, building stamina, and focusing on gradual improvements [88, 89]. To determine which forms and dose of exercise might help or exacerbate PASC, more research is necessary.

#### Sleep

##### Sleep in acute COVID-19

Sleep disorders are now recognized among the mosaic of COVID-19 symptoms. With acute COVID-19, Mass et al. found that patients with obstructive sleep apnea (OSA) had an 8-fold increased incidence of COVID-19 infection [90]. OSA was linked to an elevated risk of hospitalization and nearly doubled the likelihood of having respiratory failure in acute COVID-19. In another study, OSA was a risk factor for mortality in diabetic individuals hospitalized with COVID-19 [91].

##### Sleep in PASC

In comparison, Martimbianco et al. found between 21.7% and 53% of PASC patients to have sleep difficulties or insomnia [92]. Restless legs syndrome (RLS) is a sleep disorder that has been linked to viral infections. Weinstock et al. found that females with PASC had a 5.7% prevalence of RLS before COVID-19 and a 14.8% prevalence after COVID-19, compared to 6.7% in control females [93].

Sleep disorders appear to be an overlapping symptom in both acute COVID-19 and PASC. Further research could help determine whether primary versus secondary sleep disorders may be driving COVID-19-related sleep dysregulation and what types of behavioral and pharmacological therapies may offer benefit.

## Social determinants of COVID-19 outcomes

### Race and ethnicity

The detrimental impacts of the COVID-19 pandemic have been disproportionately felt by people of color. Recent analysis shows that the death rate for Black and Hispanic Americans is double that of Whites, taking age into account [94]. These racial disparities are consistent when comparing rates of hospitalization and infection for Black, Latinx, American Indian, Alaska Native, Asian, Native Hawaiian and Pacific Islander and other non-white racial groups with White Americans [95]. Black, Latinx, and Indigenous Americans have higher prevalence of hypertension, diabetes, and obesity, which are risk factors for PASC development [96]. Disparities are also exacerbated as racial minorities have disproportionate rates of non-COVID deaths due to lack of access to care [97].

### Barriers to health equity

Vulnerable groups face barriers to treatment that can considerably hinder management of PASC. Rates of health insurance differ considerably for Black and Latinx people compared to white people, and these disparities are particularly notable in states that did not expand Medicaid eligibility following the passing of the Affordable Care Act [95, 98]. Management of PASC could be particularly difficult for uninsured groups because healthcare costs could act as deterrents from screening and seeking advanced care [98].

Additionally, vulnerable groups may face barriers in managing PASC due to occupational and geographical factors. The PASC-related symptoms of fatigue and brain fog can interfere with work, and a lack of job security and occupational health services may hinder long-term care and daily activities [95]. Transportation barriers also prevent healthcare access, leading to missed appointments, delayed care, and poorer management of chronic illness [99]. In a survey of cancer patients in Texas, compared to 38% of whites, 55% of African Americans, and 60% of Hispanics reported poor access to transportation as a barrier to missing cancer treatment [100]. In another study, Velasco et al. found that non-English-speaking Hispanic patients were 75% more likely to require critical care than non-Hispanic patients, identifying late presentation and poor access to care as determinants of clinical outcomes [101].

### Cultural perspectives

Chronic illness, especially non-visible illness, remains poorly understood and acknowledged by the general public, leading to additional social barriers felt by PASC survivors. A study investigating the experience of patients with Chronic Fatigue Syndrome (CFS)/myalgic encephalomyelitis (ME) found that individuals often felt discredited by professionals and experienced trivialization of their illness socially and professionally, resulting in the internalization of negative feelings [102]. Validation of health conditions was found to be an important component in fostering social support and counteracting existing stigma against non-visible illness.

The pressure associated with stigmatization against non-visible illness is also readily felt in the workplace. Patients may be faced with the notion of “Damned if they do, damned if they do not” when deciding to disclose illness to employers [103]. Though laws exist to protect individuals against discrimination at work, deviant labeling and stigmatization remain deterrents for people with chronic illness. Not disclosing can lead to a lack of support and validation for the patient’s symptoms, impacting their mental and physical well-being. PASC individuals have also reported feeling invalidated by friends, family, and clinicians [104]. It is important to address the social stigma accompanying non-visible illness, and health policy interventions and further research will be necessary for educating the public on PASC and providing employment protections for patients.

### Existing biases

Though the cause of PASC has yet to be identified, the experiences of patients are undeniable.

A study involving 24 interviews with PASC patients in the UK found common themes such as difficulty managing symptoms, difficulty finding proper care, and feeling ignored and isolated by medical providers and the public. Some patients hesitated to seek care due to fears of their symptoms being dismissed as psychological rather than physical. The lack of consensus among medical professionals on treatment added to the confusion and frustration experienced by these patients [104].

PASC patients, who are predominantly women, may also face gender biases that reinforce stigma against them. Gendered norms have historically characterized men as “stoic” and women as “hysterical” while in pain, leading to health disparities such as female patients receiving sedative medication rather than analgesics for pain and waiting longer to receive treatment [105–107]. These discriminatory ideas may contribute to the misattribution of PASC symptoms to a psychiatric etiology and a failure to properly evaluate or treat PASC.

The disproportionate impact of COVID-19 on racial minorities raises concerns that PASC may be significantly underreported for underserved populations [95]. Economic, geographic, and occupational barriers may prevent these vulnerable populations from accessing proper healthcare and communicating their health concerns to clinicians. Directed screening and interventions of the demographic groups most impacted by PASC are necessary to ensure that underrepresented populations are not neglected.

## Conclusion and future directions

The emerging pattern from multiple studies suggests that acute COVID-19 and PASC affect patients with a multitude of symptoms. The varied presentations are likely influenced by patients’ age, environmental, behavioral and lifestyle risk factors. Sex-specific genetic, hormonal and immune risk factors may help explain why more men have severe outcomes from acute SARS-CoV-2 infection, while more women are affected with PASC [108]. In addition, social determinants of health are emerging as important factors in patients’ access to care and long-term outcomes.

With over 762 million people worldwide experiencing acute COVID-19 and an estimated 10–30% of the population experiencing PASC, these conditions have become critical public health concerns and ongoing research is needed to understand the evolving risk factors contributing to their presentation and progression. In addition, cross-disciplinary collaborations and federal funding are vital for research and the establishment of specialized Long-COVID clinics that can offer clinical care, rehabilitation, social work assistance, peer support groups, and equitable access to services for disadvantaged populations. It is further of utmost importance that clinicians caring for patients with Long-COVID continue to actively learn about the emerging science of Long-COVID and validate patient symptoms.

## Data Availability

Not applicable.

## List of abbreviations

SARS-CoV-2: Severe acute respiratory syndrome coronavirus 2
COVID-19: Coronavirus disease 2019
PASC: Post-acute sequelae of SARS-CoV-2 infections
EBV: Epstein-Barr Virus
HLA: Human Leukocyte Antigen
SLE: Systemic lupus erythematosus
ADT: Androgen deprivation therapy
ACE2: Angiotensin converting enzyme 2
TLR7: Toll-like receptor 7
IFN: Interferon
POTS: Postural orthostatic tachycardia syndrome
RLS: Restless legs syndrome
CFS: Chronic Fatigue Syndrome
ME: Myalgic encephalomyelitis
